# Supplementary material for: Facile synthesis of porous Mo2C/C composites by using luffa sponge-derived carbon template in molten salt media
Source: R Soc Open Sci. 2019 Jun 26;6(6):190547. doi: 10.1098/rsos.190547 (PMC6599802; doi:10.1098/rsos.190547)
Supplement: Supplementary material from "Facile synthesis of porous Mo2C/C composites by using luffa sponge-derived carbon template in molten salt media " [file rsos190547supp1.docx]

**Facile synthesis of porous Mo_2_C/C composites by using luffa sponge-derived carbon template in molten salt media**

Minzhong Huang ^a^, Wanyi Zeng * ^b^ and Ziwen zhu ^a^

*^a^Advanced Materials Lab., College of Materials, Xiamen University, No. 422 Siming South Road, Xiamen, Fujian, 361005, China.*

*^b^Institute of Urban Environment, Chinese Academy of Sciences, No. 1799 Jimei Road, Xiamen, Fujian, 361021, China.*

***Corresponding author:** Fax: +86-592-6190592; E-mail address: wyzeng@iue.ac.cn







**Figure S1.** SEM images of the MCC-800.


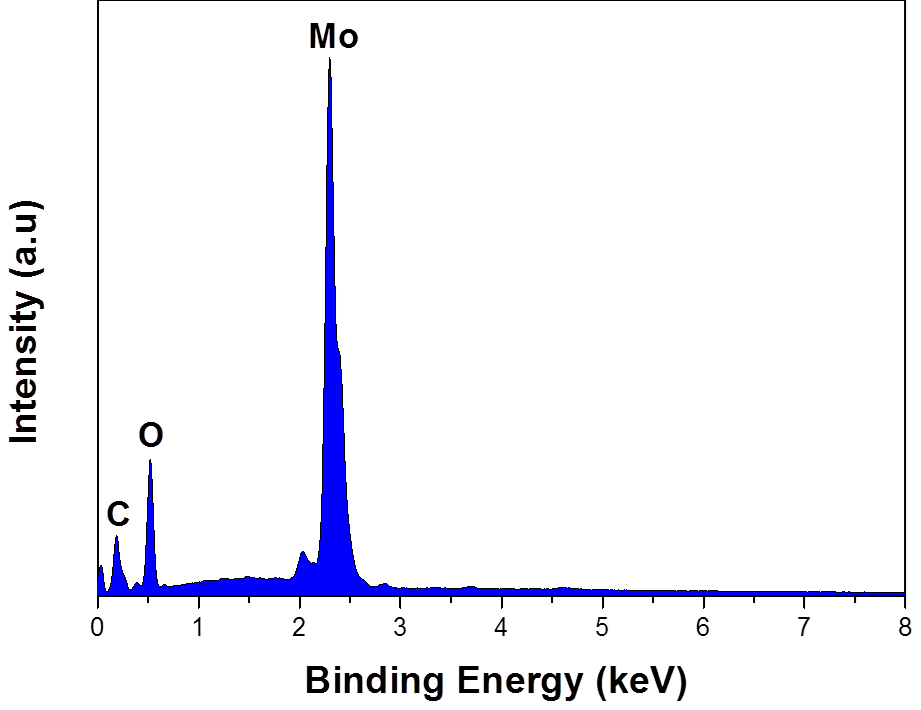


**Figure S2.** EDX spectrum of MCC-800.

**Table S1．** Specific surface area (S_BET_), total pore volume (V_total_) and average pore diameter (D_p_) of as-prepared samples.

| Sample | S_BET_ (m^2^ g^-1^) | V_total_ (cm^3^ g^-1^) | D_p_ (nm) |
| --- | --- | --- | --- |
| MCC-800 | 251.1 | 0.182 | 3.12 |


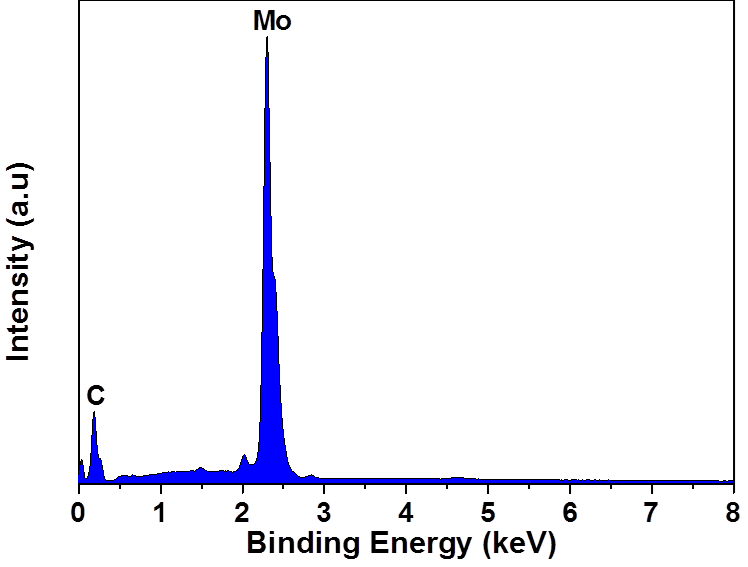


**Figure S3.** EDX spectrum of MCC-850.
